# Community-wide outbreak of haemolytic uraemic syndrome associated with Shiga toxin 2-producing *Escherichia coli* O26:H11 in southern Italy, summer 2013

**DOI:** 10.2807/1560-7917.ES.2016.21.38.30343

**Published:** 2016-09-22

**Authors:** Cinzia Germinario, Alfredo Caprioli, Mario Giordano, Maria Chironna, Maria Serena Gallone, Silvio Tafuri, Fabio Minelli, Antonella Maugliani, Valeria Michelacci, Luisa Santangelo, Onofrio Mongelli, Cosimo Montagna, Gaia Scavia, on behalf of all participants of the Outbreak investigation team

**Affiliations:** 1 Department of Biomedical Science and Human Oncology, Aldo Moro University of Bari, Bari, Italy; 2 Department of Veterinary Public Health and Food Safety, Istituto Superiore di Sanità, Rome, Italy; 3 Paediatric Nephrology and Dialysis Unit, Children's Hospital Giovanni XXIII, Bari, Italy; 4 Veterinary Public Health Office, Regione Puglia, Bari, Italy; 5 Istituto Zooprofilattico Sperimentale della Puglia e Basilicata, Putignano, Italy; 6 The members of the Outbreak investigation team are listed at the end of the article

**Keywords:** Shiga toxin-producing Escherichia coli, Vero cytotoxin-producing E. coli, Haemolytic Uraemic Syndrome, Epidemiology, Disease outbreak, Surveillance: Outbreak investigation, Molecular epidemiology, Italy

## Abstract

In summer 2013, an excess of paediatric cases of haemolytic uraemic syndrome (HUS) in a southern region of Italy prompted the investigation of a community-wide outbreak of Shiga toxin 2-producing *Escherichia coli* (STEC) O26:H11 infections. Case finding was based on testing patients with HUS or bloody diarrhoea for STEC infection by microbiological and serological methods. A case–control study was conducted to identify the source of the outbreak. STEC O26 infection was identified in 20 children (median age 17 months) with HUS, two of whom reported severe neurological sequelae. No cases in adults were detected. Molecular typing showed that two distinct STEC O26:H11 strains were involved. The case–control study showed an association between STEC O26 infection and consumption of dairy products from two local plants, but not with specific ready-to-eat products. *E.coli* O26:H11 strains lacking the *stx* genes were isolated from bulk milk and curd samples, but their PFGE profiles did not match those of the outbreak isolates. This outbreak supports the view that infections with Stx2-producing *E. coli* O26 in children have a high probability of progressing to HUS and represent an emerging public health problem in Europe.

## Introduction

Haemolytic uraemic syndrome (HUS) is a rare disorder characterised by microangiopathic haemolysis, platelet consumption, and multi-organ damage (mainly to the kidneys) [1]. In its typical form, it occurs after a prodromal diarrhoea, usually due to Shiga toxin (Stx)-producing *Escherichia coli* (STEC) infection. HUS is the most common cause of acute renal failure in childhood and occurs in ca 15% of children with STEC O157 infections [1]. Although STEC O157 is the predominant cause of paediatric HUS worldwide [1], cases associated with infections with STEC belonging to non-O157 serogroups have been increasingly reported [2-5].

In Italy, surveillance of HUS in children (< 15 years) was established in 1988 through the National Registry of HUS, carried out by the Italian Society for Paediatric Nephrology in cooperation with the National Reference Laboratory for *E. coli* [4]. Between 1988 and 2012, an average of 33 sporadic cases of HUS per year were observed in Italy, with a mean annual incidence of 0.4 cases per 100,000 residents aged 0–15 years. The STEC serogroups most frequently reported were O157 (35%), O26 (26%), O145 (12%), O111 (10%) and O103 (5%) [6].

### The outbreak

Between 4 June and 9 August 2013, seven paediatric cases of HUS resident in the Apulia region (19,345 km^2^, ca 4 million inhabitants) or with a history of recent travel to the area were reported to the National Registry of HUS. This represented an excess with respect to the three to five cases per year annually reported in the Apulia region since 1988 [4], and laboratory investigation showed evidence of infection with STEC O26 for four of the five cases whose clinical samples were examined. An outbreak limited to the Apulia region was suspected and an alert was issued to the regional health authorities. Investigations were started to find additional cases, identify the sources of infection, and limit the spread of the outbreak. This report describes the epidemiological, clinical, and microbiological features of the outbreak.

## Methods

### Case definition and case finding

A probable case was defined as a patient presenting with HUS, or with suspected HUS, or with bloody diarrhoea between 1 June and 30 September 2013, resident in, or with a history of travelling to the Apulia region during the 15 days before the onset of illness. A confirmed case was defined as a patient with diarrhoea or HUS, and laboratory evidence of infection with STEC O26. HUS cases were defined according to Tozzi et al. [4] as patients with evidence of renal failure, intravascular haemolysis, and thrombocytopenia (platelet count < 100,000/mm^3^).

In the Apulia region, the active case finding was carried out by alerting hospitals and emergency rooms to promptly report to the regional surveillance system for infectious diseases any case of bloody diarrhoea, or HUS, or suspected HUS and to submit stool and serum samples for the laboratory diagnosis of STEC infection. The case-finding was extended at the national and international level by posting alerts through the dedicated information systems coordinated by the National HUS Registry and the European Centre for Disease Prevention and Control (ECDC).

### Laboratory diagnosis of Shiga toxin 2-producing *Escherichia coli* infection

Stool samples were inoculated in buffered peptone water (BPW) and incubated at 37 °C for 18 hours. DNA was extracted from 1 mL of the culture with the InstaGene Matrix (Bio-rad Laboratories, Hercules, CA, US) and tested by a real-time PCR assay to detect the presence of Stx- (*stx1* and *stx2*) [7] and intimin (*eae*)-coding genes [8]. PCR-positive samples were streaked onto MacConkey agar plates and colonies resembling *E. coli* tested for the presence of *stx* and *eae* genes by PCR [9]. The *stx* and/or *eae*-positive strains were tested with O antisera against the main STEC serogroups (Statens Serum Institut, Copenhagen, Denmark) by slide agglutination. Serotyping of STEC belonging to other serogroups was kindly performed by F. Scheutz, at the Statens Serum Institut, Copenhagen, Denmark. Stools were also examined for the presence of free Stx by the Vero cell cytotoxicity assay [4]. Serum samples were tested for antibodies to the lipopolysaccharide (LPS) of five major STEC serogroups (O157, O26, O103, O111, and O145) by ELISA [10].

### Characterisation of the Shiga toxin 2-producing *Escherichia coli* O26 strains

The flagellar (H-antigen) *fliC* alleles were detected by real-time PCR, as described by Madic et al. [11]. The *stx* gene subtyping was carried out by PCR, as described by Scheutz et al. [12]. PFGE (Pulsed-Field Gel Electrophoresis) was performed as previously described [9]. Similarity of PFGE profiles was evaluated using the Bionumerics software (Applied Maths, Sint-Martens-Latem, Belgium), using the UPGMA algorithm with tolerance and optimisation set at 1.5%.

Multilocus sequence typing (MLST) was performed using the scheme developed by Wirth et al. [13]. Sequence types (STs) were determined using the tool available at the University of Warwick [13].

### Epidemiological investigation

Parents of confirmed cases were interviewed using the HUS Registry questionnaire, after giving their informed consent. Age, sex, type and time of onset of clinical symptoms, and food and environmental exposures for STEC infection in the 7 days before illness onset were annotated. Other information included dietary habits, food and water consumption, exposure to livestock, presence of household contacts with diarrhoea, exposures to potential environmental sources of STEC, and travel in the 2 weeks before the onset of symptoms.

### Food trace-back investigation

Trace-back investigations were mainly focused on the retail outlets that sold dairy products and vegetables to the families of the cases in the two weeks before the onset of symptoms. Based on the interviews, it was hypothesised that these products might be implicated in the transmission of STEC infection to patients. Indeed, they were the only items consumed by most of the cases, for which the mode of preparation and consumption would not have eliminated any possible contamination with STEC.

The identified dairy plants were inspected and their staff interviewed by local health authorities regarding processing practices and sources of raw materials. The dairy farms that supplied milk to the plants were identified and visited as well. Samples of ready-to-eat dairy products, curd, raw milk, fruit and vegetables, and ground beef were collected and tested for the presence of STEC O26 according to the ISO/TS 13136:2012 method for the detection of STEC in food. Briefly, 25 g of each sample were enriched in BPW at 37 °C for 18h. DNA was extracted from one mL of the enrichment culture with the InstaGene Matrix and tested by real-time PCR for the presence of *stx1*, *stx2*, and *eae* genes. Positive DNA samples were further tested for the *wzx* gene associated to the O26 *E. coli* antigen [7]. Positive enrichment cultures were subjected to an O26-specific immunomagnetic separation followed by plating on MacConkey Agar. Colonies were tested for the presence of *stx* and/or *eae* genes by PCR amplification [9].

### Environmental investigation

Marine water samples were collected from seaside locations that had been attended by some of the cases. Water samples (250 mL) were subjected to filtration through membranes of mean pore size 0.45 μm. Membranes were then transferred to BPW for enrichment at 37 °C for 18h. DNA extraction and real-time PCR assays for STEC virulence genes were carried out as described for stool and food samples.

### Case–control study

A case–control study, limited to the 15 laboratory confirmed cases resident in the Apulia region, was conducted to identify exposures associated with STEC O26 infection in the 10 days before illness onset. Controls, up to five for each case, were children who were reported not to have had diarrhoea in the month preceding the interview, randomly selected among the patients of the family paediatricians of the cases. Controls were matched by sex, age and area of residence. Exposures possibly associated with STEC infection and reported by cases during the hypothesis generation were investigated. Cases and controls were interviewed face-to-face and by telephone, respectively. Exposures associated with STEC O26 infection were analysed by exact univariate and multivariate logistic regression. For subjects reporting consumption of dairy products, univariate and multivariate analyses were carried out to identify associations between STEC O26 infection and the dairy plant of origin of the artisan products of bovine origin. The level of significance was set at p < 0.05. Data were analysed by Stata 11 MP (StataCorp LP, College Station, Texas).

## Results

### Case finding and diagnosis of Shiga toxin 2-producing *Escherichia coli* infection

**Figure 1:**
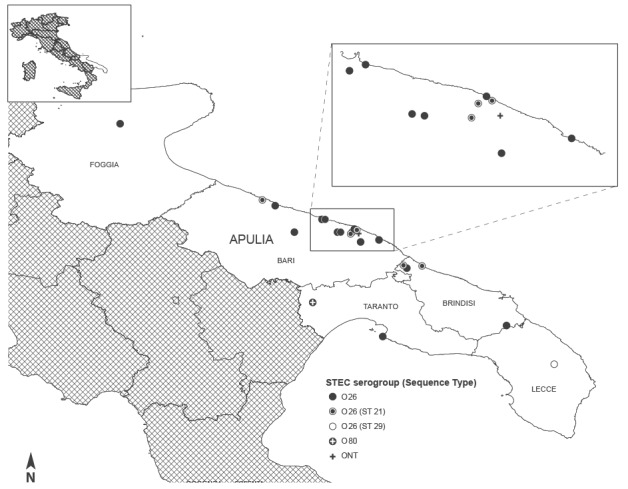
Geographical distribution of recorded cases of haemolytic uraemic syndrome, Apulia region, Italy, 1 June to 30 September 2013 (n=22)

ONT: O non-typeable; STEC: Shiga toxin-producing *Escherichia coli*.

Between 1 June and 30 September 2013, 17 children with HUS resident in the Apulia region were admitted to the regional paediatric nephrology centre participating in the National HUS Registry. Two of the 17 cases were siblings and fell ill 10 days apart. The active case-finding revealed five additional children with HUS with a history of travel to the Apulia region: four were diagnosed in other Italian hospitals and one in a Swiss hospital. Figure 1 shows a map of the Apulia region with the location of the HUS cases at the onset of prodromal symptoms.

Stool and serum samples were obtained from 19 HUS cases, and serum only from the remaining three. The mean interval between onset of enteric symptoms and stool collection was 9 days (range 5–14 days). Stool examination yielded the isolation of STEC O26:H11 strains positive for the *stx2a* and *eae* genes from seven cases. STEC strains belonging to serotypes O80:H2, positive for *stx2f* and *eae*, and O non-typeable (NT) positive for *stx2* group and *eae* were isolated from two other cases. Four other cases had the enrichment cultures positive for *stx2* genes in PCR and/or had free faecal Stx. Serum antibodies against the LPS of *E. coli* O26 were detected in 20 cases. The two patients with STEC belonging to other serogroups did not have antibodies to any of the LPS tested (Table 1). For the six cases with serum antibodies against the LPS of *E.coli* O26 and STEC and Stx-negative faeces, the mean interval between onset of symptoms and stool collection was 10.5 days, longer than that of cases with Stx-positive stools (7 days). Overall, the evidence of infection with STEC O26 was obtained for 20 cases: 16 were resident in the Apulia region and four were travel-related. STEC O26 was isolated from five residents and two travel-related cases.

**Table 1:** Evidence of Shiga toxin-producing *Escherichia coli* infection in 22 cases admitted to hospital with haemolytic uraemic syndrome between 1 June and 30 September 2013 and resident in or with a history of travel to Apulia region, Italy in the 15 days before illness onset

Laboratory diagnosis: Type of evidence	No. of cases
**Infection with STEC O26 (confirmed cases):**	**20**
**Isolation of *E. coli* O26:H11 (*stx2a* + , *eae* + ) and O26 LPS antibodies**	**7**
Free faecal Stx and/or *stx2* genes, and O26 LPS antbodies: 4 cases	4
O26 LPS antibodies only	9
**Infection with other STEC:**	**2**
Isolation of *E. coli* O80:H2 (*stx2f* + , *eae* + )	1
Isolation of *E. coli* ONT (*stx2* group + , *eae* + )	1

LPS: lipopolysaccharide; ONT: O non-typeable; STEC: Shiga toxin 2-producing *Escherichia coli*; Stx: Shiga toxin.

The active case finding also allowed the identification of 20 cases of bloody diarrhoea whose stools were negative for salmonella and campylobacter and were examined for the presence of STEC. A STEC O157 strain was isolated from one of these patients while the faeces of the other 19 were negative for *stx* and *eae* genes.

Stool samples were also obtained from 26 of 40 household contacts of 12 confirmed cases. No STEC strains were isolated, but one sample collected by an adult and one from a child, both presenting with gastroenteric symptoms, tested positive in PCR for the *stx*2 and the *eae* gene, respectively.

**Figure 2:**
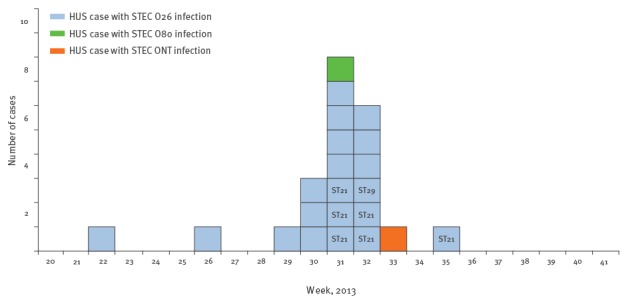
Distribution of cases of haemolytic uraemic syndrome by infection with different Shiga toxin-producing *Escherichia coli* strains, Apulia region, Italy, 1 June to 30 September 2013 (n=22)

HUS: haemolytic uraemic syndrome; ONT: O non-typeable; STEC: Shiga toxin 2-producing *Escherichia coli*.

In the 20 confirmed cases with STEC O26 infection, the onset of symptoms occurred between 4 June and 8 September 2013 (Figure 2).

Boxes represent HUS cases and are coloured according to the different STEC O serogroups. Where available, the sequence type (ST) of the infecting STEC O26 strain is indicated in the box.

### Case characteristics

The age of the 20 confirmed cases ranged from 11 to 78 months (mean: 24 months; median: 17 months). Eleven cases were female. All confirmed cases developed HUS. Prodromal diarrhoea was reported for 18 cases, and in 10 of them it was bloody. The median interval between onset of diarrhoea and diagnosis of HUS was 9 days (range 1–20 days). Vomiting, abdominal pain, and fever (> 38 °C) were reported for 16, 9, and 6 cases, respectively. Other clinical details were available for 19 children. On admission, six cases presented neurological symptoms, primarily seizures (n = 5). Seven cases had haematuria and 10 cases had oliguria or anuria. Four cases underwent haemodialysis and plasma exchange, and four patients haemodialysis only. Blood transfusions were administered to 17 patients and 13 also received plasma infusion. Severe neurological sequelae as of 18 months after the onset of HUS were reported for two cases. One of these children also presented a light chronic renal failure.

### Source hypothesis generation

Parents of children were interviewed to generate hypotheses about sources of infection but no obvious link was identified that could explain a significant number of them. Of the 19 confirmed cases that could be interviewed, four had household members with diarrhoea in the 15 days before the onset of illness and six reported contact with farm animals. As for food exposure, of the 18 patients for which the information was available, most cases reported consumption of cooked bovine meat (78%), pasteurised or UHT milk (61%), yoghurt (72%), artisanal dairy products (mainly ricotta and mozzarella cheese) of bovine origin (100%), fresh fruit (61%) and watermelon (83%), with multiple brands and/or food retailers involved. Interviews also revealed that two seaside beaches were attended by two and four cases, respectively.

### Trace-back investigations and laboratory examination of food and environmental samples

The dairy products of bovine origin consumed by 16 confirmed cases had been prepared by at least six different plants (Table 2). These plants were inspected between 20 August 2013 and 13 September 2013, as well as other 14 dairy plants supplying the retail outlets attended by the cases’ families. Recommendations for implementing hygiene measures and good manufacturing practices were issued. The activity of one plant showed gross hygiene failures and was suspended. Pasteurisation of milk for dairy product production was prescribed to three other plants. Visits were also carried out to 31 dairy farms that supplied raw milk to the plants. A total of 218 samples of raw milk and dairy products of bovine origin were collected and tested for the presence of STEC. Sixty-five samples of fruit and vegetables, in particular watermelon, and five beef samples were also collected at retail and wholesale outlets identified through the trace-back investigations and tested for STEC. All the fruit, vegetable, and beef samples were negative. The enrichment cultures of 12 bulk milk or curd samples were positive for *stx* genes and, in 10 samples, for the *eae* and *wzx_O26_* genes. *E. coli* O26:H11 strains lacking the *stx* genes but positive for the *eae* gene were isolated from two curd and two bulk milk specimens (Table 2).

**Table 2:** Presence of *Escherichia coli* O26 and/or its virulence genes in bulk milk or curd samples collected in the Apulia region, Italy, 20 August to 13 September 2013

Dairy plant^a^	Confirmed cases associated with the dairy plant	Number and type of samples associated with the dairy plants				
		Samples positive for *stx*, *eae* and *wzx_O26_* genes		Samples with *E. coli O26* isolation		
	**n**	**n**	**Type of matrix**	**n**	**Type of matrix**	**Characteristics of the strain**
A	3	3	1 curd, 2 bulk milk^b^	1	curd	*E. coli* O26 *stx*-, *eae* +
B	2	1	bulk milk^b^	0	0	0
C	7	0	0	0	0	0
D	2	0	0	0	0	0
E	1	6	4 curd, 2 bulk milk	2	milk, curd	*E. coli* O26 *stx*-, *eae* +
F	1	1	bulk milk	1	milk	*E. coli* O26 *stx*-, *eae* +
Other dairy farms	Not possible to determine	2	bulk milk^c^	0	0	0
Total	16	12	5 curd, 7 bulk milk^c^	4	2 milk, 2 curd	*E. coli* O26 *stx*-, *eae* +

E. coli: Escherichia coli.

^a^ Samples were collected directly at the plant or at dairy farms supplying milk to the plant.

^b^ One bulk milk sample was from a farm that supplied both plant A and B.

^c^ Two samples were positive for *stx* genes only.

Samples were from dairy plants linked with confirmed cases of Shiga toxin-producing *Escherichia coli *O26 infection or dairy farms supplying milk to the plants.

Fifteen marine water samples collected at the two seaside beaches attended by the cases proved negative for the presence of *stx* and *eae* genes.

### Characterisation of the *Escherichia coli* O26 strains

**Figure 3:**
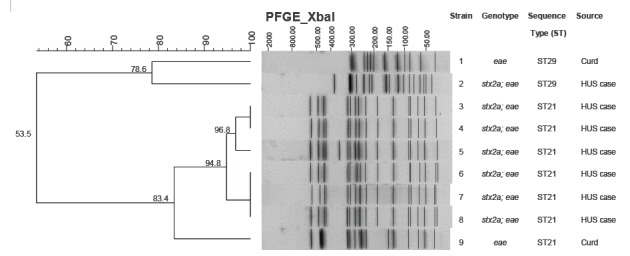
Dendrogram of the degree (%) of similarity between pulsed-field gel electrophoresis profiles of XbaI-digested genomic DNA from strains of *Escherichia coli* O26 isolated from cases of haemolytic uraemic syndrome and dairy products, Apulia region, Italy, 1 June and 30 September 2013

The Stx-positive and Stx-negative *E. coli* O26 strains isolated respectively from cases and from dairy products were characterised by MLST and PFGE. All the strains belonged to the clonal complex ST29, but two different STs were distinguished: six strains from cases and one strain from food belonged to ST21, while one human and three food strains were ST29. PFGE analysis (Figure 3) of the strains isolated from cases showed similar profiles (between 94.8% and 100% similarity) for the six ST21 strains and a clearly different profile for the ST29 strain. The PFGE profiles of the two Stx-negative strains isolated from curd were not related with the two profiles of the human strains, and the two strains from milk were untypable due to degradation of DNA during the procedure.

### Case–control study

Fifteen confirmed cases and 52 matched controls were included in the case–control study. No differences in the mean age (t = 0.2; p = 0.80) and in the distribution by sex (Chi-squared = 0.01; p = 0.91) were observed between cases and controls. In the univariate analysis (Table 3), STEC O26 infection was significantly associated with the consumption of products from dairy plants A and C but was associated neither with any individual dairy product from these plants nor with any other food items. Multivariate analysis confirmed the association for both plant A (odds ratio (OR): 42.7; 95% confidence interval (CI):2.4–750.5; p = 0.01) and plant C (OR:21.3; 95% CI:3.0–152.5; p < 0.01).

**Table 3:** Results of the case–control analysis of exposures in an outbreak of Shiga toxin-producing *Escherichia coli* O26 infection, Apulia region, Italy, 2013

Exposure	Cases (n = 15)	Controls (n = 52)	Matched OR (95% CI)	*p*-value
Meat				
Chicken	11	33	1.6 (0.4–7.7)	0.5
Frankfurter sausages	4	8	2 (0.4–9.2)	0.3
Hamburger	4	21	0.6 (0.1–2.8)	0.4
Meatballs	4	19	0.5 (0.1–2.4)	0.4
Pork	5	19	0.9 (0.2–3.3)	0.8
Sausages	5	26	0.5 (0.1–1.9)	0.3
Veal	12	28	3.1 (0.7–19.2)	0.1
Fruit and vegetables				
Fresh fruit	8	32	0.7 (0.2–2.7)	0.6
Green leafy vegetables	3	4	2.9 (0.4–19.3)	0.2
Fruit juice	8	30	0.8 (0.2–3.2)	0.8
Watermelon	12	39	1.3 (0.3–8.5)	0.7
Other vegetables	7	22	1.2 (0.3–4.4)	0.8
Dairy products				
Pasteurised milk	5	15	1.2 (0.3–4.8)	0.7
UHT milk	4	24	0.4 (0.1–1.6)	0.2
Yogurt	11	31	1.9 (0.5–9.0)	0.3
Burrata cheese	3	5	2.4 (0.3–13.9)	0.3
Mozzarella cheese	8	22	1.6 (0.5–5.9)	0.5
Ricotta cheese	8	32	0.7 (0.2–2.6)	0.5
Other fresh cheeses	3	28	0.2 (0.0–0.9)	0.2
Any dairy product from				
Plant A	3	1	10.3 (1.5–930.2)	< 0.01
Plant B	2	5	1.4 (0.1–10.1)	0.7
Plant C	7	5	13.9 (2.2–43.4)	< 0.01
Plant E	1	2	1.8 (0.0–36.3)	0.6
Other food				
Ice cream	10	41	0.5 (0.1–2.5)	0.3

CI: confidence interval; OR: odds ratio; UHT: ultra-high temperature processing.

Results are from the univariate analysis.

## Discussion

This report describes the largest outbreak of STEC-associated HUS ever observed in Italy. The STEC serotype involved was O26:H11, which represents the most common cause of STEC non-O157 infections in Europe [2,14] and has been frequently associated with HUS worldwide [3,4,15-18]. In Italy, the proportion of HUS cases associated with STEC O26 infection has increased since the late 1990s, and currently exceeds that of STEC O157 [2].

This particular STEC serogroup seems to be evolving [5], with a shift from the *stx*1 to the *stx2* genotype in the strains associated with severe illness that occurred over the last two decades [2,5,19]. Such a phenomenon has public health relevance, since Stx2-producing *E. coli* O26 strains can cause a disease that is as severe as that caused by STEC O157 [15,19-22]. Two of the cases involved in this episode reported severe neurological sequelae.

At the time of writing this report, to our knowledge, the episode herein described represented the second community-wide outbreak of HUS caused by Stx2-producing *E. coli* O26 after that involving 16 HUS cases in France in 2005, linked to the consumption of unpasteurised cow’s cheese (camembert) [16,23]. Another severe community-wide outbreak of HUS mainly associated with STEC O26 infection was been reported in Romania in early 2016, with at least 15 children involved [24].

In the Italian outbreak, as in other STEC community-wide outbreaks [10,25-28], cases occurred over a large geographic area and a prolonged period of time and were observed in the framework of a HUS surveillance system, confirming that the emergence of HUS clusters represents an important sentinel event for outbreak recognition [2,10,25,26]. In this episode, an active case finding was promptly implemented after the alert to find new cases, improving the sensitivity and the promptness of case reporting. The enhanced regional surveillance system facilitated the outbreak investigation as well as the adoption of public health measures. Moreover, the existence of national HUS surveillance systems allowed a timely finding of STEC O26 cases resident in other Italian regions and in Switzerland, and who had visited Apulia.

A prompt and accurate laboratory diagnosis of STEC infection is of the utmost importance in HUS cases, to assess the STEC serotype/genotype involved. In 13 out of the 20 cases involved in this episode, the diagnosis of STEC O26 infection was based only on the detection of LPS antibodies, confirming the importance of LPS serology in identifying STEC O157 and non-O157 infections in HUS patients [4,10,16]. Molecular typing of the seven STEC O26:H11 isolates from cases showed that two distinct STEC O26 strains were involved in the outbreak, with the one belonging to ST21 playing a major role. The concomitant presence of two different STEC O26:H11 strains and the two cases of HUS due to different STEC serotypes in the same area and period of time suggest the possibility of a multiple-aetiology outbreak [29]. Outbreaks with different non-O157 STEC strains, including STEC O26, have been reported in the United States [29], Belgium [30] and France [16,23]. As in our case, one of them involved two STEC strains belonging to serotypes O26:H11 and O80:H2 [16,23] and another one, two STEC O26 strains displaying different PFGE profiles [29].

Multiple-aetiology outbreaks have been frequently associated with exposures to environmental sources [29]. Some of the cases shared exposure to the same seaside locations, but water samples collected at those places proved negative for STEC, although the size of the samples may have been too small to allow the detection of the pathogen. However, in this outbreak the spread of cases in a large geographic area makes environmental sources unlike.

Although the origin of human infections with Stx2-producing *E. coli* O26 strains has rarely been identified [31], at least two episodes involving cases of HUS and associated with consumption of unpasteurised milk or dairy products have been reported in Austria [32] and France [16,23]. In our investigation, STEC O26 infection was significantly associated with the consumption of dairy products from two local plants and a drop in the occurrence of cases was observed after the adoption of control measures involving those plants. Neither the association of STEC O26 infection with specific products nor a laboratory evidence of STEC contamination in the final ready-to-eat dairy products could be demonstrated. However, we cannot exclude that a contaminated raw material with a prolonged shelf life, such as a frozen ingredient, could have been continuously used in local plants to prepare different fresh, ready-to-eat products, even though no evidence of such use emerged from the visits to the dairy plants.

The possible involvement of dairy products was also suggested by the evidence of STEC contamination in some bulk milk and curd samples from different plants and by the isolation of *stx*-negative *E. coli* O26:H11 strains from four of these samples. The loss and transfer of *stx* genes by *E. coli* O26 has been demonstrated during human infections [33], and Stx2-positive and negative variants of the same STEC O26:H11 strain, as defined by PFGE analysis, have been isolated from both HUS cases and cheese samples in the French camembert-associated outbreak [23]. Conversely, in the present episode the PFGE profiles of the *stx*-negative *E. coli* O26:H11 strains from milk and curd did not match those of the outbreak isolates.

Another interesting feature of this outbreak is that, despite the enhanced surveillance, we were unable to identify cases of STEC O26 infection other than young children with HUS. In outbreaks due to STEC O157, conversely, severe diarrhoea is generally observed in all the age bands, with young children being more prone to develop HUS [1,25]. The apparent absence of adult cases of infection could be explained by a lack of exposure to the source of infection, even though the epidemiological investigation did not show any suspect food dedicated to small children or other possible STEC risk factors restricted to the young. Another possibility is that the outbreak source was contaminated at a very low level, albeit sufficient to cause disease in young children, the most susceptible age group. Stx2-producing *E. coli* O26 is considered a highly virulent STEC [5]. Nonetheless, an analysis of the literature confirms that the reported outbreaks [16,32] and severe cases [15,19-22] have generally involved only young children. Moreover, in HUS surveys [4], children with STEC O26 infection have been reported to be younger than those with STEC O157 infection. Altogether, these observations allow us to speculate that Stx2-producing *E. coli* O26 might exert a particular virulence towards young children, which could be the reflection of a better fitness of these pathogens in a particular intestinal environment, eventually resulting in an increased colonisation of the gastrointestinal tract.

In conclusion, the present outbreak supports the view that infections with Stx2-producing *E. coli* O26 in children have a high probability to progress to HUS and represent an emerging public health problem in Europe [5]. This further underlines the importance of maintaining national and local surveillance systems for HUS for an early detection and response to STEC O26 infections.

## Notes

**Members of the Outbreak investigation team:** Other participants of the outbreak investigation team: Francesco Desiante, Giulia Calabrese, Carmen Martino, Maria Filomena Gallone, Anna Morea (Department of Biomedical Science and Human Oncology, Aldo Moro University of Bari, Italy), Rosangela Tozzoli, Stefano Morabito (Department of Veterinary Public Health and Food Safety, Istituto Superiore di Sanità, Rome, Italy), Antonio Parisi (Istituto Zooprofilattico Sperimentale della Puglia e Basilicata, Putignano, Italy), Tommaso Depalo, Vincenza Carbone, Annalisa Resta (Paediatric Nephrology and Dialysis Unit, Children's Hospital Giovanni XXIII, Bari, Italy), Pierfrancesco Pinto (Veterinary Public Health Office, Regione Puglia, Bari, Italy ), Massimo Ricci (Regional Agency for Prevention and Environment Protection, Brindisi, Italy), Alessandra Gianviti (Children’s Hospital Bambino Gesù, Rome, Italy), Gianluigi Ardissino (IRCCS Ospedale Maggior Policlinico, Clinica De’ Marchi, Milan, Italy ).
